# Anti-Obesity Effect of Theabrownin from Dark Tea in C57BL/6J Mice Fed a High-Fat Diet by Metabolic Profiles through Gut Microbiota Using Untargeted Metabolomics

**DOI:** 10.3390/foods11193000

**Published:** 2022-09-27

**Authors:** Hang-Yu Li, Si-Yu Huang, Ruo-Gu Xiong, Si-Xia Wu, Dan-Dan Zhou, Adila Saimaiti, Min Luo, Hui-Lian Zhu, Hua-Bin Li

**Affiliations:** Guangdong Provincial Key Laboratory of Food, Nutrition and Health, Department of Nutrition, School of Public Health, Sun Yat-Sen University, Guangzhou 510080, China

**Keywords:** dark tea, theabrownin, anti-obesity, gut microbiota, metabolomics

## Abstract

The epidemic of obesity is a serious public health problem. In this study, the effect of theabrownin from dark tea on obesity was evaluated by biochemical tests and nuclear magnetic resonance in C57BL/6J mice fed a high-fat diet. A mixture of antibiotics was used to deplete gut microbiota and then fecal microbiota transplant was used to restore gut microbiota. Untargeted metabolomics was used to reveal the effects of theabrownin on metabolic profiles through gut microbiota. The results showed that theabrownin significantly reduced body weight gain (83.0%) and body fat accumulation (30.29%) without affecting appetite. Also, theabrownin promoted lipid clearance with a hepatoprotective effect. The extra antibiotics disrupted the regulation of theabrownin on weight control while fecal microbiota transplant restored the beneficial regulation. That is, gut microbiota was important for theabrownin to reduce body weight gain. The untargeted metabolomics indicated that 18 metabolites were related to the anti-obesity effect of theabrownin mediated by gut microbiota. Furthermore, phenylalanine metabolism, histidine metabolism, as well as protein digestion and absorption pathway played a role in the anti-obesity of theabrownin. Our findings suggested that theabrownin significantly alleviated obesity via gut microbiota-related metabolic pathways, and theabrownin could be used for the prevention and treatment of obesity.

## 1. Introduction

Obesity has presented as a serious threat to public health [1]. It has been estimated that there could be 206 million children and adolescents suffering from obesity in 2025, and the number could reach up to 254 million in 2030 [1]. Obesity is characterized by the accumulation of body fat and the increase in body weight, and is considered to be a risk factor for cardiovascular diseases, stroke, type 2 diabetes mellitus, and hypertension [2]. Moreover, the accumulation of body fat can increase the metabolic burden on liver [3]. The overconsumption of high-calorie foods is one of the major causes of obesity, and gut microbial dysbiosis could play a role in the pathogenesis of obesity [4,5]. According to the epidemiological evidence, the supplement of healthy plant-based foods and their bioactive components in the daily diet is inversely related to the development of obesity (relative risk (RR) = 0.31, 95% confidence interval (95% CI): 0.12–0.77) [6]. Furthermore, tea (*Camellia sinensis*), a popular healthy beverage, possesses an excellent potential to alleviate obesity [7].

Tea is classified into six categories according to the degree of fermentation, among which dark tea is a post-fermented tea produced by a microorganism-involved special piling fermentation process [8]. Dark tea has been reported to possess multiple beneficial properties, such as cardiovascular protective, anti-diabetic, anti-obesity, and hepatoprotective effects [9]. In our previous studies, dark tea could promote lipid clearance by regulating gut microbiota [10,11]. Due to the unique fermented process, dark tea has organoleptic characteristics and bioactive components that are distinct from other teas [9]. During the fermentation of dark tea, tea polyphenols are markedly decreased, while theabrownin (TB) is significantly increased and can reach up to 7–13% of dark tea weight [9,12,13,14]. A standardized and industrialized process has been established to extract and isolate TB, which is based on many steps of liquid–liquid extraction and precipitation processes using different organic solvents [15,16,17]. TB is safe and well-tolerated in animal models; to be specific, the oral median lethal dose (LD_50_) of TB for mice is greater than 10,000 mg/kg body mass [18,19,20]. In previous studies, TB with low and medium dosages showed a certain anti-obesity effect (30–40% reduction of body weight gain), and gut microbiota like *Clostridium* could play a role in it [19,21,22]. However, whether the intervention of TB at a higher dosage had a significant anti-obesity effect is not known, which is worth assessing because TB is safe and well-tolerated at a very high dosage.

In this study, we evaluated the effect of TB with a high dosage on obesity in mice fed a high-fat diet (HFD), and the results showed that TB significantly reduced body weight gain by 83.0%. This is much higher than previously reported in the literature (the 30–40% reduction of body weight gain from TB intervention with 125–1125 mg/kg/day) [19,20,21,22,23]. Traditional biochemical tests were used to assess the changes in the indexes of lipid metabolism. Nuclear magnetic resonance was used to accurately evaluate the accumulation of body fat. Extra antibiotics and fecal microbiota transplant were used to change the gut microbial community for further evaluating metabolic profiles related to gut microbiota via untargeted metabolomics.

## 2. Materials and Methods

### 2.1. Materials

Theabrownin (TB) was provided by Yunnan Tangren Biotechnology Co., Ltd. (Honghe, Yunnan, China), and its molecular weight range is 0.4 kDa–50 kDa with characteristic absorption at the wavelength of 380 nm. Vancomycin, ampicillin, metronidazole, and neomycin were purchased from Sigma-Aldrich (St. Louis, MO, USA).

### 2.2. Animal Experimental Design

The male four-week-old specific-pathogen-free (SPF) C57BL/6J mice were purchased from the Guangdong medical laboratory animal center (Guangzhou, China) and housed in the SPF animal facility (22–24 °C, 50–60% relative humidity, and a 12-h light/dark cycle). The C57BL/6J mice were fed either a control diet (CD) (D12450J, Research Diet, New Brunswick, NJ, USA) or a high-fat diet (HFD) (D12492, Research Diet, New Brunswick, NJ, USA). All experiments in this study followed the guidelines of the laboratory animal center at Sun Yat-Sen University (Guangzhou, China), and all procedures were approved by the Ethics Committee of the School of Public Health, Sun Yat-Sen University (No. 2019-002).

**Figure 1:**
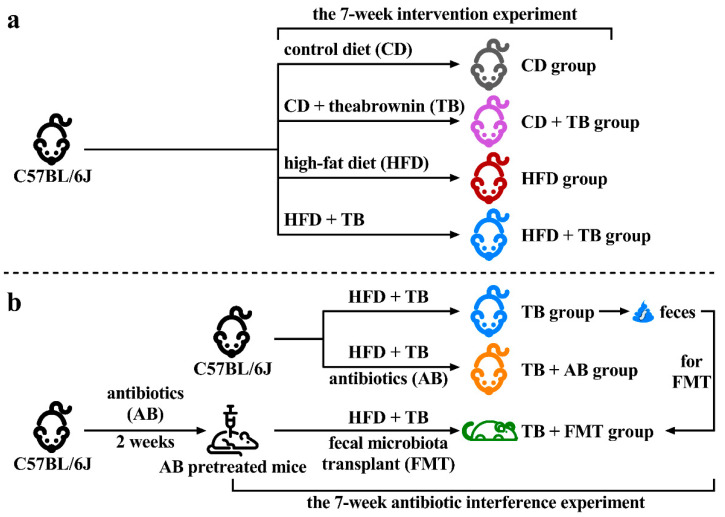
Simplified flow chart of grouping and experimental design. (**a**) The design of the 7-week intervention experiment. (**b**) The design of the 7-week antibiotic interference experiment. Abbreviation: AB, antibiotics; CD, control diet; FMT, fecal microbiota transplant; HFD, high-fat diet; TB, theabrownin.

In the 7-week intervention experiment, 20 C57BL/6J mice were randomly divided into four groups: a group of mice received CD as blank control (CD group), a group of mice received CD and TB (CD + TB group), a group of mice received HFD to induce obese model (HFD group), the last group of mice received HFD and TB (HFD + TB group). In the 7-week antibiotic interference experiment, 15 C57BL/6J mice were randomly divided into three groups: a group of C57BL/6J mice received HFD and TB (TB group). A group of C57BL/6J mice simultaneously received HFD, TB, and antibiotics (TB + AB group). The last group of C57BL/6J mice was pretreated with antibiotics to deplete original gut microbiota, and then received HFD, TB, and fecal microbiota transplant (TB + FMT group). The TB was dissolved in sterilized water and provided to mice by gavage with a dosage of 2300 mg/kg/day. The antibiotics contained 0.5 mg/mL of vancomycin and 1.0 mg/mL of ampicillin, metronidazole, and neomycin. The feces from the TB group were collected to prepare fecal microbial supernatant for transplantation, and the antibiotics were added to drinking water [24]. The body weight and feces were recorded and collected. The blood and liver were collected after mice were euthanized. The blood sample was clotted and then the serum was isolated by centrifugation with 2000 rpm for 15 min. The simplified flow chart of the experimental design is exhibited in Figure 1.

### 2.3. Fecal Microbiota Transplant

The mice in the TB group were selected as the donors of feces. Referring to a previous study, 200 mg of fresh feces were collected and blended in 1 mL of 37 °C sterilized PBS on the day of the transplantation [25]. After centrifuging at 1500 rpm/min for 5 min, the supernatant was collected. Then, the extra antibiotics were terminated and 300 μL of the supernatant was provided to the antibiotics-pretreated mice by gavage. During the first five days, the fecal microbiota was transplanted once a day, then the transplantation was repeated once a week for the remaining seven weeks (Figure 1).

### 2.4. Blood Biochemical Tests and Nuclear Magnetic Resonance

According to the manufacturers’ protocols, the commercial kits (Nanjing Jiancheng Bioengineering Institute, Nanjing, China) and the automatic biochemical analyzer (Chemray 800, Rayto, Shenzhen, China) were used to measure the biochemical indexes of serum, such as triglyceride (TG), high-density lipoprotein cholesterol (HDL-C), low-density lipoprotein cholesterol (LDL-C), alanine aminotransferase (ALT), and aspartate aminotransferase (AST). Liver tissue was homogenized in cold saline (1:10, m/v) and then centrifuged (3000 rpm/min for 10 min) to collect the supernatant for measuring hepatic TG level. Body fat distribution and body composition involving body fat rate and muscle mass were measured with a nuclear magnetic resonance analyzer (QMR23-040H-I, Niumag Analytical Instrument Corporation, Suzhou, China).

### 2.5. Liver Histopathology

The examination of hepatic injury included hematoxylin and eosin (H&E) staining as well as Oil red O staining. The H&E and Oil red O staining were conducted with regular procedures. The 4% paraformaldehyde and paraffin were used to fix and embed liver tissues, and then 5-μm thick slices were sectioned for H&E staining. Other parts of liver tissues were frozen in optimal cutting temperature compound (OCT, 4583, SAKURA, Tokyo, Japan), and then 10-μm thick slices were sectioned for Oil red O staining. The images of H&E and Oil red O staining were obtained on light microscopy (Eclipse Ci-L, Nikon, Tokyo, Japan), microscope camera (DS-Fi2, Nikon, Tokyo, Japan), and digital pathology slides scanner (KF-PRO-120, KFBio, Ningbo, China).

### 2.6. Untargeted Metabolomics

The 400 µL of methanol (−20 °C) was mixed with a 100 µL serum sample and vortexed for 60 s. Under 4 °C, the mixture was centrifuged at 12,000 rpm/min for 10 min to collect the supernatant which was further concentrated to dryness in a vacuum. Then, the powder of the sample was dissolved in 150 µL 80% methanol solution containing 2-chlorobenzalanine (4 ppm). Next, the resuspended solution was filtered by a 0.22 µm membrane to prepare the sample for liquid chromatography-mass spectrometry analysis (LC-MS). Meanwhile, a part of the sample was used to conduct quality control.

For LC separation, the Thermo Vanquish system equipped with an ACQUITY UPLC HSS T3 column (150 × 2.1 mm, 1.8 μm, Waters, Waltham, MA, USA) was used. The column was maintained at 40 °C, and the temperature of the autosampler was 8 °C. For the positive mode, the mobile phase consisted of 0.1% formic acid in water (A2) and 0.1% formic acid in acetonitrile (B2). For the negative mode, the mobile phase consisted of 5 mM ammonium formate in water (A3) and pure acetonitrile (B3). The flow rate was 0.25 mL/min, and 2 μL of the sample was injected into the system after equilibration. An increasing linear gradient of solvent B2/B3 (*v/v*) was used as follows: 0–1 min, 2% B2/B3; 1–9 min, 2–50% B2/B3; 9–12 min, 50–98% B2/B3; 12–13.5 min, 98% B2/B3; 13.5–14 min, 98–2% B2/B3; 14–20 min, 2% B2-positive mode (14–17 min, 2% B3-negative mode).

The Thermo Q Exactive mass spectrometer (Waltham, MA, USA) was used for subsequent electrospray ionization tandem mass spectrometry experiments (ESI-MSn). The spray voltage was 3.5 kV and −2.5 kV for positive and negative modes, respectively. The sheath gas was 30 arbitrary units. The auxiliary gas was 10 arbitrary units. The capillary temperature was 325 °C. The analyzer scanned over a mass range of the mass-to-charge ratio (*m/z*, 81 to 1000) for a full scan at a mass resolution of 70,000. Data-dependent acquisition (DDA) MS/MS experiments were conducted with higher energy collision-induced dissociation (HCD) scan, and the normalized collision energy was 30 eV. To filter unnecessary information in MS/MS spectra, dynamic exclusion was used. Metabolites were identified according to information from databases such as the human metabolome database (HMDB), METLIN, MassBank, LipidMaps, mzCloud, as well as the self-developed metabolomic database of PANOMIX Biomedical Tech (Suzhou, China).

### 2.7. Statistical Analysis

The statistical analysis was conducted with SPSS software (version 26.0, IBM, Armonk, NY, USA). The data were shown as mean ± standard deviation (SD) or median with interquartile range according to the normality test. The analysis of variance (ANOVA) was used when the datasets had normality and homoscedasticity, otherwise, the Kruskal–Wallis test would be used. Figures were generated by GraphPad Prism (version 9.0.0, GraphPad Software, San Diego, CA, USA) and Origin software (version 2022, OriginLab, Northampton, MA, USA). The raw data of metabolomics were converted to the mzXML format by Proteowizard (Proteowizard software, Palo Alto, CA, USA), and the R package XCMS was used for further data processing, including the identification, filtration, and alignment of peaks. Orthogonal partial least squares discriminant analysis (OPLS-DA) was used to detect the differences in metabolites among groups and generate the “variable importance for the projection” (VIP value). Permutation test was used to validate the OPLS-DA model with the R^2^ and Q^2^ values. The differential metabolites were determined by the *p*-value of multivariate statistical analysis <0.05 and VIP > 1. The metabolic pathway enrichment analysis referred to the Kyoto encyclopedia of genes and genomes databases (KEGG).

## 3. Results

### 3.1. Effects of Theabrownin (TB) on Body Weight and Appetite

**Figure 2:**
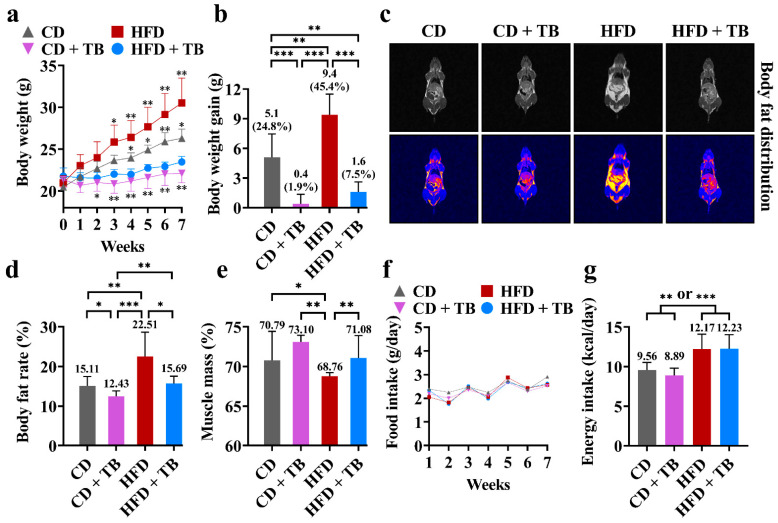
The effects of TB on obesity. (**a**) The trend of body weight change. (**b**) Body weight gain at the end of the experiment. (**c**) The images of body fat distribution with or without pseudo color by nuclear magnetic resonance scan (the bright light parts are adipose tissue). (**d**) The body fat rate at the end of the experiment. (**e**) The muscle mass rate at the end of the experiment. (**f**) Average food intake per mouse. (**g**) Average energy intake per mouse. * *p*-value < 0.05, ** *p*-value < 0.01, *** *p*-value < 0.001. Abbreviation: CD, control diet; HFD, high-fat diet; TB, theabrownin.

The changes in body weight and body fat during the intervention experiment are shown in Figure 2. The HFD group showed an increased trend of body weight gain compared with the CD group (Figure 2a). The intervention of TB reduced the body weight gain in the CD + TB group and especially in the HFD + TB group (Figure 2a,b). Compared with the original body weights at the beginning of the experiment, the body weights increased by 24.8%, 1.9%, 45.4%, and 7.5% in the CD, CD + TB, HFD, and HFD + TB groups, respectively (Figure 2b). That is, the body weight gain in the HFD + TB group was decreased by 83.0% compared with the HFD group. It should be pointed out that TB also showed the inhibition effect on body weight gain in the CD + TB groups, which meant TB with high dosage might be more suitable for patients with obesity, but not people with normal weight. Moreover, the images from nuclear magnetic resonance showed that TB alleviated body fat accumulation when the HFD + TB group was compared with the HFD group (Figure 2c). Furthermore, the assessment of body composition via nuclear magnetic resonance showed that the body fat rate in the HFD group was 48.97% higher than that of the CD group, whereas the body fat rate in the HFD + TB group was 30.29% lower than that of the HFD group (Figure 2d). Additionally, TB preserved muscle mass during the promotion of significant weight loss and lipolysis (Figure 2e). TB did not affect the appetite of mice in the CD + TB and HFD + TB groups compared with the CD and HFD groups, respectively (Figure 2f,g). In short, TB reduced body weight gain and body fat accumulation and protected muscle mass without affecting appetite.

### 3.2. Effects of TB on Hepatic Indexes

**Figure 3:**
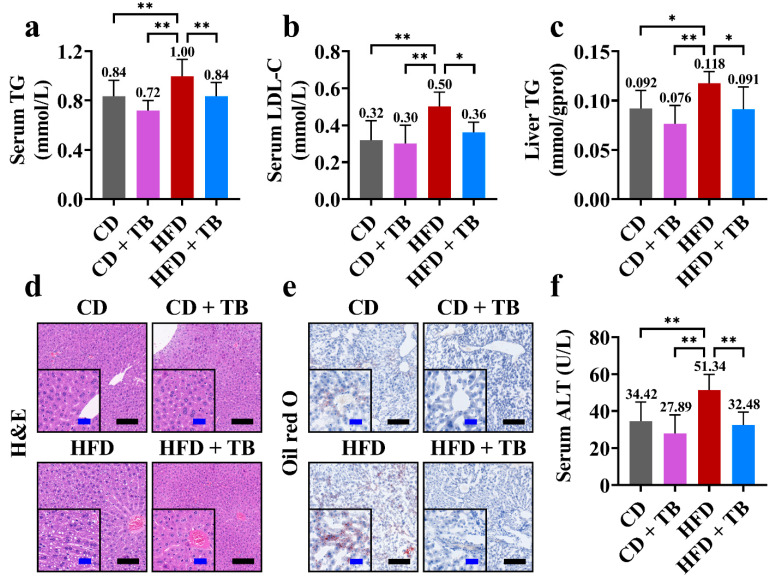
The effects of TB on lipid metabolism and hepatoprotection. (**a**) The serum level of TG. (**b**) The serum level of LDL-C. (**c**) The hepatic level of TG. (**d**) The hepatic H&E staining (black scale bar = 200 μm and blue scale bar = 50 μm). (**e**) The hepatic Oil red O staining (black scale bar = 200 μm and blue scale bar = 50 μm). (**f**) The serum level of ALT. * *p*-value < 0.05, ** *p*-value < 0.01. Abbreviation: ALT, alanine aminotransferase; CD, control diet; H&E, hematoxylin and eosin; HFD, high-fat diet; LDL-C, low-density lipoprotein cholesterol; TB, theabrownin, TG, triglyceride.

The changes in hepatic indexes during the intervention experiment are shown in Figure 3. The HFD group showed a significant increase in the serum level of TG compared with the CD group, whereas the HFD + TB group showed a significant decrease in the serum level of TG compared with the HFD group (Figure 3a). Similarly, the serum level of LDL-C was increased in the HFD group while the serum level of LDL-C was decreased in the HFD + TB group (Figure 3b). Moreover, TB promoted lipid clearance and alleviated the metabolic burden of lipid metabolism in liver, which were reflected in decreasing the hepatic level of TG, the formation of hepatic lipid drops, and hepatic lipid accumulation (Figure 3c–e). Additionally, the serum level of ALT in the HFD + TB group was lower than that of the HFD group (Figure 3f). Furthermore, there was no sign of liver injury between the CD + TB group and the CD group (Figure 3d–f). HDL-C and AST showed no significant changes among groups (data not shown). These findings indicated that TB could safely promote lipid clearance and release the pressure of hepatic lipid metabolism with a hepatoprotective effect.

### 3.3. TB, Gut Microbiota, and Body Weight

**Figure 4:**
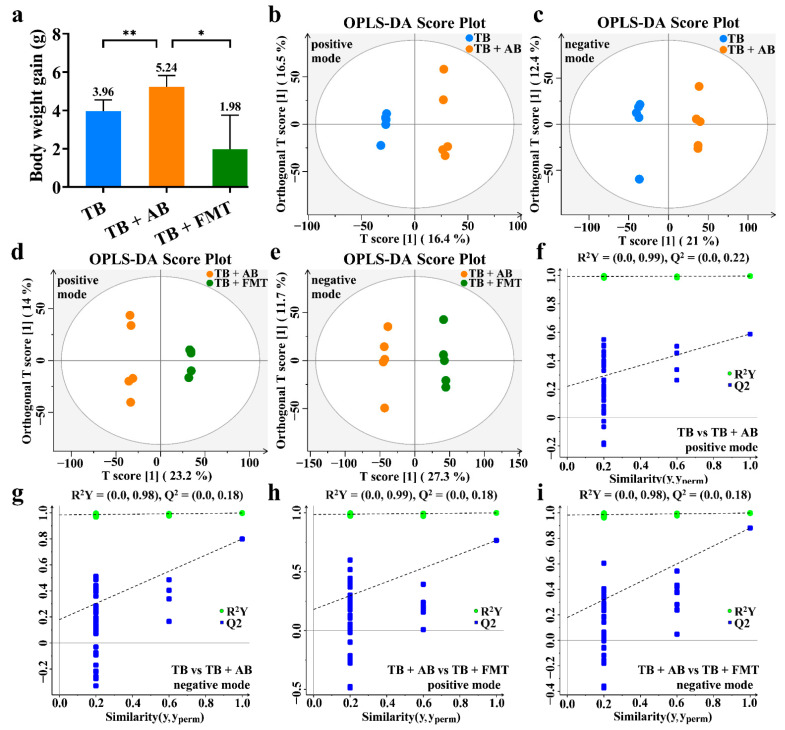
The body weight gain and the OPLS-DA of the untargeted metabolomics. (**a**) Body weight gain. (**b**) The OPLS-DA between the TB and TB + AB groups in the positive mode. (**c**) The OPLS-DA between the TB and TB + AB groups in the negative mode. (**d**) The OPLS-DA between the TB + AB and TB + FMT groups in the positive mode. (**e**) The OPLS-DA between the TB + AB and TB + FMT groups in the negative mode. (**f**) The permutation of OPLS-DA between the TB and TB + AB groups in the positive mode. (**g**) The permutation of OPLS-DA between the TB and TB + AB groups in the negative mode. (**h**) The permutation of OPLS-DA between the TB + AB and TB + FMT groups in the positive mode. (**i**) The permutation of OPLS-DA between the TB + AB and TB + FMT groups in the negative mode. Abbreviation: AB, antibiotics; FMT, fecal microbiota transplant; OPLS-DA, orthogonal partial least squares discriminant analysis; TB, theabrownin. * *p*-value < 0.05, ** *p*-value < 0.01.

The extra supplement of antibiotics (AB) was widely used to induce gut microbial dysbiosis, while the transplantation of fecal microbiota (FMT) was commonly used to restore gut microbial homeostasis [24,25]. In the antibiotic interference experiment, the TB + AB group had a worse body weight control than the TB group, and the body weight gain in the TB + AB group showed a 32.3% higher than that in the TB group (Figure 4a). On the other hand, the TB + FMT group had better body weight control than the TB + AB group, and the body weight gain in the TB + FMT group showed 62.2% lower than that in the TB + AB group (Figure 4a). That is, gut microbiota could be important for TB to reduce body weight gain.

### 3.4. The OPLS-DA among the TB, TB + Antibiotics, and TB + Fecal Microbiota Transplant Groups

We further conducted untargeted metabolomics among the TB, TB + AB, and TB + FMT groups to evaluate the gut microbiota-related metabolic profiles in obese mice under the intervention of TB. As shown in Figure 4b,c, the OPLS-DA score plots revealed significant clustering trends of metabolites between the TB and TB + AB groups in the positive mode (R^2^X = 0.329, R^2^Y = 0.996, Q^2^ = 0.588) as well as in the negative mode (R^2^X = 0.334, R^2^Y = 0.998, Q^2^ = 0.790). As shown in Figure 4d,e, the significant differences in metabolites were also discovered between the TB + AB and TB + FMT groups in the positive mode (R^2^X = 0.372, R^2^Y = 0.999, Q^2^ = 0.781) as well as in the negative mode (R^2^X = 0.390, R^2^Y = 0.998, Q^2^ = 0.867). The results of the permutation tests for the OPLS-DA are exhibited in Figure 4f–i. These results implied that there were significant differences in metabolites between the TB and TB + AB groups as well as the TB + AB and TB + FMT groups.

### 3.5. The Identification of Differential Metabolites in the Untargeted Metabolomics

**Figure 5:**
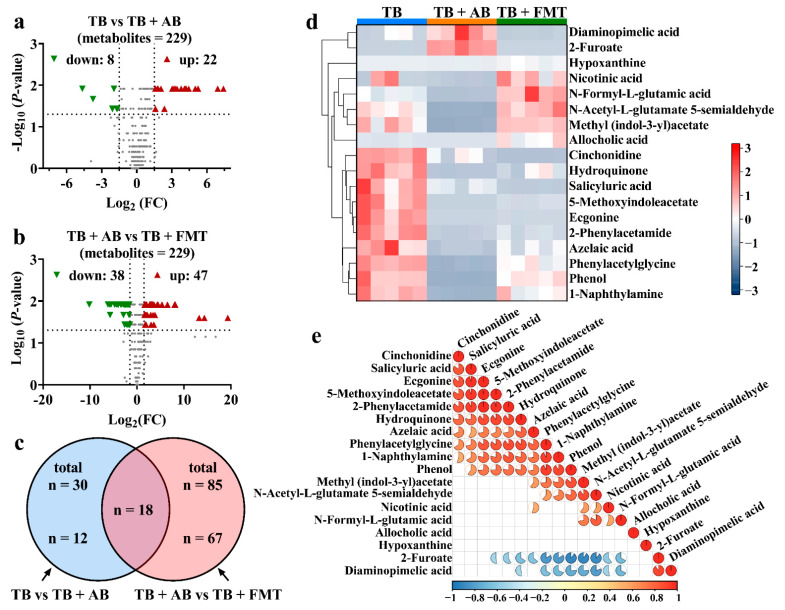
The overall changes in metabolic profiles and representative metabolites. (**a**) The changes in metabolites between the TB and TB + AB groups. (**b**) The changes in metabolites between the TB + AB and TB + FMT groups. (**c**) The cross-comparative analysis of metabolites among groups. (**d**) The heatmap of the 18 representative metabolites. (**e**) The correlation analysis among the 18 representative metabolites (the complete circle corresponds to a correlation coefficient (*r*) = 1 or −1). Abbreviation: AB, antibiotics; FC, fold change; FMT, fecal microbiota transplant; TB, theabrownin.

Following the clues from the OPLS-DA, 1808 metabolites were identified based on the m/z and retention time, among which 472 metabolites were further confirmed according to the information from ion fragments. Next, 229 metabolites showed significant differences among the TB, TB + AB, and TB + FMT groups. Further, there were 30 metabolites with a significant fold change (FC) between the TB and TB + AB groups, and there were 85 metabolites with a significant FC between the TB + AB and TB + FMT groups (Log_2_ FC > 1.5 or < −1.5, and *p*-value < 0.05) (Figure 5a,b). Moreover, the 18 metabolites were simultaneously contained in the dataset of 30 metabolites and the dataset of 85 metabolites (Figure 5c). The changes in the 18 representative metabolites are exhibited in Figure 5d. For example, the serum levels of 2-phenylacetamide, phenylacetylglycine, N-Formyl-L-glutamic acid, and phenol were decreased in the TB + AB group compared with the TB group, whereas the serum levels of these metabolites were increased in the TB + FMT group compared with the TB + AB group (Figure 5d). Some of the 18 representative metabolites were significantly correlated to each other (Figure 5e). For example, 2-phenylacetamide was positively correlated to phenylacetylglycine (*r* = 0.89, *p*-value < 0.001) and phenol (*r* = 0.77, *p*-value < 0.001), meanwhile, phenylacetylglycine was positively correlated to phenol (*r* = 0.93, *p*-value < 0.001). These results revealed the representative metabolites (such as 2-phenylacetamide, phenylacetylglycine, N-Formyl-L-glutamic acid, and phenol) could be involved in the weight control function of TB mediated by gut microbiota.

### 3.6. The Evaluation of Metabolic Pathways

**Figure 6:**
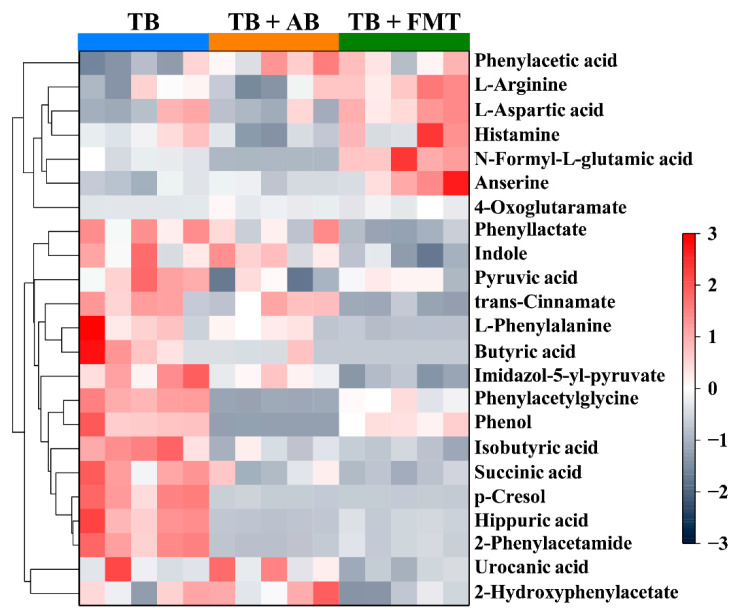
The changes in contents of metabolites which were involved in the three common metabolic pathways. Abbreviation: AB, antibiotics; FMT, fecal microbiota transplant; TB, theabrownin.

We further analyzed the changes in metabolic pathways based on 229 differential metabolites to better understand the gut microbiota-related mechanisms of TB in alleviating obesity. As shown in Table 1, there were 12 metabolic pathways significantly changed between the TB and TB + AB groups (*p*-value < 0.05). Meanwhile, there were 11 metabolic pathways significantly changed between the TB + AB and TB + FMT groups (*p*-value < 0.05). After cross-comparing the 12 metabolic pathways with the 11 metabolic pathways, several metabolic pathways were the same, such as phenylalanine metabolism, histidine metabolism, as well as protein digestion and absorption pathway. Moreover, some of the 18 representative metabolites (such as 2-phenylacetamide, phenylacetylglycine, N-Formyl-L-glutamic acid, and phenol) were involved in these 3 metabolic pathways (Table 2). The changes of metabolites involved in the 3 common metabolic pathways are exhibited in Figure 6. In short, phenylalanine metabolism, histidine metabolism, as well as protein digestion and absorption pathway could be important contributors to TB to reduce body weight gain through gut microbiota.

## 4. Discussion

The content of TB was significantly increased while tea polyphenols (such as catechins and flavonoids) were reduced during the fermentation of dark tea [26]. Previous studies based on mouse and rat models had described the bioactivities of TB with a dosage from 100 mg/kg/day to 1280 mg/kg/day [15,17,18,19,20,21,22,23,27,28]. For example, Pu-erh tea (a typical dark tea) had a certain effect on body weight control, meanwhile, 225 mg/kg/day of TB could alleviate hypercholesterolemia, evidenced using 3-week-old male C57BL/6J mice [17]. Additionally, 225 mg/kg/day of TB could prevent adipogenesis in 4-week-old C57BL/6J mice [21]. Moreover, 1124 mg/kg/day of TB showed a certain effect on preventing body weight gain in rats [19]. That is, previous studies have focused on the effects of TB with a relative low dosage, especially when a mouse model was used [17,21,22,27]. Previous studies have orally administrated TB to rats at a dosage around 1100–1200 mg/kg/day which was well tolerated, and according to the difference in body surface area in different animal models, the effective dose for mice is twice of that for rats [15,18,19,20,23,28,29]; therefore, in this study, a dosage of 2300 mg/kg/day TB was used. Moreover, in this study, nuclear magnetic resonance analyzer was used to accurately evaluate the changes in body fat distribution and provide precise data of body fat rate under the intervention of TB at a higher dosage. The results showed that a relatively short-term (7 weeks) intervention of TB significantly reduced body weight gain (83.0%) and body fat accumulation (30.29%) without affecting appetite. Furthermore, TB markedly alleviated obesity and protected muscle mass (Figure 2). A prospective study reported that the loss of body weight could accompany the loss of muscle mass and strength, which might have adverse effects on physical function and metabolism [30]. Furthermore, an *in vivo* study showed that the reduced muscle performance of obesity would not reverse after weight loss [31]. Therefore, TB could be an ideal substance to overcome obesity while preserving muscle mass. Besides, body fat accumulation is often accompanied by the metabolic burden in liver [32,33]; in this study, TB released the pressure of lipid clearance in liver and showed a hepatoprotective effect (Figure 3). In short, TB could safely achieve an ideal anti-obesity effect in a relatively short time.

Gut microbial dysbiosis is considered to be a risk factor for obesity, while, on the other hand, the bioactive activity of dark tea and TB could be related to gut microbiota [34]. Under the intake of TB, gut microbiota-related metabolic profiles in the obese model have not been evaluated. In this study, the extra intake of multiple antibiotics (a conventional method to induce gut microbial dysbiosis [35,36]) disrupted the regulation of TB on body weight control, while fecal microbiota transplant recovered the beneficial regulation (Figure 4). That is, TB could reduce body weight gain through gut microbiota. Further untargeted metabolomics showed that some metabolites (such as 2-phenylacetamide, phenylacetylglycine, N-Formyl-L-glutamic acid, and phenol), as well as their corresponding metabolic pathways (such as phenylalanine metabolism, histidine metabolism, and protein digestion and absorption pathway), were significantly changed, followed by the alteration of gut microbiota (Figure 5, Table 1 and Table 2). Several studies have reported that phenylalanine metabolism was related to obesity [37]. For example, the obese patient could suffer from abnormal phenylalanine metabolism, and the in vivo study showed that several molecules involved in phenylalanine metabolism might be predictors of the development of obesity [38,39,40]. Moreover, the regulation of phenylalanine metabolism disorder could accompany the alleviation of obesity [41]. Besides, histidine metabolism was also related to obesity [42]. Several clinical studies showed that histidine metabolism was related to the richness of gut microbiota, and it was involved in the pathophysiology of obesity by affecting iron status via gut microbiota [43,44]. Additionally, protein is the fundamental macronutrient for maintaining life. The digestion and absorption of protein could directly regulate energy homeostasis and indirectly affect lipid metabolism through gut microbiota [45,46,47]. In summary, our findings uncovered the changes in gut microbiota-related metabolic profiles in obesity under the intake of TB.

## 5. Conclusions

In this study, the effects of TB on obesity have been evaluated by metabolic profiles through gut microbiota using nuclear magnetic resonance and untargeted metabolomics. The results suggest that TB possesses a favorable anti-obesity effect (reducing 83.0% of body weight gain) while preserving muscle mass, and gut microbiota plays a vital role in it. Moreover, phenylalanine metabolism, histidine metabolism, and protein digestion and absorption pathway could be involved in the gut microbiota-dependent mechanisms of TB to alleviate obesity. This paper focused on the anti-obesity effect of TB with high dosage, as well as metabolic profiles through gut microbiota, which completed the missing puzzle among previous studies. On the other hand, other anti-obesity mechanisms (like the possible synergistic or antagonistic effects between TB and other components of tea on gut microbiota) should be studied in the future. In addition, clinical trials are needed to verify the effect of TB on obese patients.

## Figures and Tables

**Table 1:** The changes in metabolic pathways among groups.

Groups	Metabolic Pathways	Total	Hits	*p*-Value	Impact
TB vs. TB + AB	Alanine, aspartate, and glutamate metabolism	28	3	0.024287	0.071023
	Caffeine metabolism	22	3	0.012576	0.14706
	Central carbon metabolism in cancer	37	4	0.009072	0.15094
	Citrate cycle (TCA cycle)	20	4	0.00088982	0.13333
	Glucagon signaling pathway	26	4	0.0024774	0.20455
	HIF-1 signaling pathway	15	2	0.043847	0.26316
	Histidine metabolism	47	4	0.020681	0.144
	Phenylalanine metabolism	60	6	0.0020825	0.10714
	Protein digestion and absorption	47	4	0.020681	0.085106
	Pyrimidine metabolism	65	5	0.014892	0.13541
	Pyruvate metabolism	31	3	0.031759	0.14286
	Tyrosine metabolism	78	7	0.0016704	0.072
TB + AB vs. TB + FMT	ABC transporters	138	10	0.042268	0.072464
	Arginine biosynthesis	23	5	0.0016344	0.18239
	beta-Alanine metabolism	32	5	0.0073705	0.17241
	Caffeine metabolism	22	5	0.0013201	0.20588
	Central carbon metabolism in cancer	37	6	0.0027667	0.15094
	Histidine metabolism	47	5	0.035216	0.112
	Pentose phosphate pathway	35	4	0.046187	0.059701
	Phenylalanine metabolism	60	7	0.0082768	0.17857
	Phenylalanine, tyrosine, and tryptophan biosynthesis	34	4	0.042145	0.13158
	Protein digestion and absorption	47	6	0.0092762	0.12766
	Tryptophan metabolism	83	12	0.0000713	0.13897

Abbreviation: AB, antibiotics; FMT, fecal microbiota transplant; TB, theabrownin.

**Table 2:** The common metabolic pathways between the TB and TB + AB groups as well as the TB + AB and TB + FMT groups.

Pathways	Total	Hits	Compounds
Phenylalanine metabolism	60	10	2-Hydroxyphenylacetate (0.75, 3.09) ^#^, 2-Phenylacetamide * (37.54, 0.19), Hippuric acid (16.80, 0.39), L-Phenylalanine (1.79, 4.00), Phenylacetic acid (0.82, 1.05), Phenylacetylglycine * (9.73, 0.19), Phenyllactate (1.15, 1.29), Pyruvic acid (2.15, 0.68), Succinic acid (1.90, 1.57), trans-Cinnamate (1.16, 4.05)
Histidine metabolism	47	7	4-Oxoglutaramate (0.26, 0.21), Anserine (0.75, 0.35); Histamine (1.65, 0.47), Imidazol-5-yl-pyruvate (1.57, 4.00), L-Aspartic acid (1.66, 0.32), N-Formyl-L-glutamic acid * (8.98, 0.04), Urocanic acid (0.84, 2.11)
Protein digestion and absorption	47	9	Butyric acid (3.74, 9653.20), Histamine (1.65, 0.47), Indole (1.01, 1.20), L-Arginine (1.18, 0.49), L-Aspartic acid (1.66, 0.32), L-Phenylalanine (1.79, 4.00), Isobutyric acid (3.03, 1.53), p-Cresol (8.16, 1.10); Phenol * (38.05, 0.04)

Abbreviation: AB, antibiotics; FMT, fecal microbiota transplant; TB, theabrownin. ^#^ The values of parentheses represent the ratio of peak area between TB group and TB + AB group, as well as the ratio of peak area between TB + AB group and TB + FMT group. * One of the 18 representative metabolites.

## Data Availability

The data presented in this study are available within the article.
